# Halogenated Thermally Activated Delayed Fluorescence Materials for Efficient Scintillation

**DOI:** 10.34133/research.0090

**Published:** 2023-03-27

**Authors:** Xiao Wang, Guowei Niu, Zixing Zhou, Zhicheng Song, Ke Qin, Xiaokang Yao, Zhijian Yang, Xiaoze Wang, He Wang, Zhuang Liu, Chengzhu Yin, Huili Ma, Kang Shen, Huifang Shi, Jun Yin, Qiushui Chen, Zhongfu An, Wei Huang

**Affiliations:** ^1^ The Institute of Flexible Electronics (IFE, Future Technologies), Xiamen University, Xiamen 361005, China.; ^2^ Key Laboratory of Flexible Electronics (KLOFE) and Institute of Advanced Materials (IAM), Nanjing Tech University, Nanjing 211816, China.; ^3^ MOE Key Laboratory for Analytical Science of Food Safety and Biology, State Key Laboratory of Photocatalysis on Energy and Environment, College of Chemistry, Fuzhou University, Fuzhou 350108, China.; ^4^ Frontiers Science Center for Flexible Electronics (FSCFE), MIIT Key Laboratory of Flexible Electronics (KLoFE), Northwestern Polytechnical University, Xi'an 710072, China.; ^5^ State Key Laboratory of Organic Electronics and Information Displays & Institute of Advanced Materials (IAM), Nanjing University of Posts and Telecommunications,9 Wenyuan Road, Nanjing 210023, China.; ^6^ Department of Applied Physics, The Hong Kong Polytechnic University, Kowloon 999077 Hong Kong, China.

## Abstract

Organic scintillators, materials with the ability to exhibit luminescence when exposed to X-rays, have aroused increasing interest in recent years. However, the enhancement of radioluminescence and improving X-ray absorption of organic scintillators lie in the inherent dilemma, due to the waste of triplet excitons and weak X-ray absorption during scintillation. Here, we employ halogenated thermally activated delayed fluorescence materials to improve the triplet exciton utilization and X-ray absorption simultaneously, generating efficient scintillation with a low detection limit, which is one order of magnitude lower than the dosage for X-ray medical diagnostics. Through experimental study and theoretical calculation, we reveal the positive role of X-ray absorption, quantum yields of prompt fluorescence, and intersystem crossing in promoting the radioluminescence intensity. This finding offers an opportunity to design diverse types of organic scintillators and expands the applications of thermally activated delayed fluorescence.

## Introduction

Scintillators have caught increasing attention among science parallel technology owing to their great potential in radiation detection and biomedical applications [1–15]. Strong absorption of X-rays is a prerequisite for achieving efficient scintillators, which makes most of the scintillators limited in heavy-metal-containing materials so far [11,16,17], since the X-ray absorption is proportional to the 4th power of the atomic number (attenuation coefficient *μ* ∝ *Z*^4^) [18–20]. However, the high preparation temperature, scarce resources, and potential toxicity to the environment and human bodies of these materials may limit their practical applications. Metal-free organic scintillators have natural advantages such as milder preparation conditions, good mechanical flexibility, and large-area fabrication [19,21,22]. For this reason, metal-free organic scintillators are good candidates for scintillation. A mass of organic scintillators with fluorescence features has been developed in the past decades [1,23]. However, the composed light atoms and low exciton utilization efficiency of traditional organic fluorescence scintillators hit a bottleneck in this field.

**Fig. 1.**
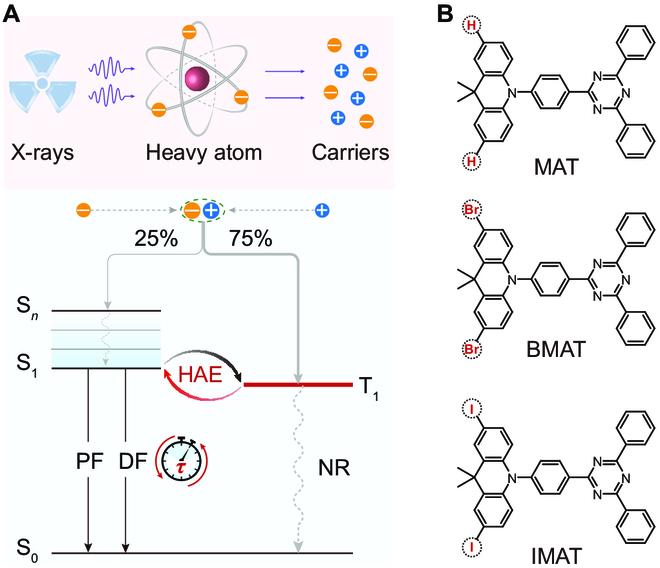
Schematic representation of the TADF-based metal-free organic scintillators. (A) Typical mechanism of TADF scintillators under exposure of X-rays. X-rays excite mainly heavy atoms via Compton scattering and the photoelectric effect, generating massive high-energy carriers including electrons and holes. After thermalization, the electrons and holes recombine to produce 25% singlet excitons and 75% triplet excitons. Compared with classical fluorescent scintillators, TADF-based scintillators can harness triplet excitons by RISC, which also benefits from the heavy atom effect (HAE). Subsequently, the generated massive singlet excitons give birth to PF and DF, improving the performance of RL. Meanwhile, heavy atoms are beneficial for decreasing the lifetimes of emission. PF, prompt fluorescence; DF, delayed fluorescence; NR, nonradiative decay. (B) Molecular structures of the TADF materials.

Notably, heavy atoms can be employed as a solution to improve the X-ray absorption ability [24–26]; however, the heavy atoms can generally facilitate the intersystem crossing (ISC) process [27] and enhance the nonradiative decay rate of singlet excitons, which impairs the fluorescence. That is, the enhanced ISC populates triplet excitons, which normally show dark-state characteristic, quenching the fluorescence [28]. Therefore, taking full advantage of triplet excitons is essential for developing efficient organic scintillators [29–34]. Thermally activated delayed fluorescence (TADF) represents a useful strategy to harvest triplet excitons [32,35–51], as triplet excitons can undergo spin conversion to singlet excitons via reverse ISC (RISC) (Fig. 1A). Considering this, halogenated TADF materials render themselves proper for exploiting metal-free organic scintillators. Besides, introducing heavy atoms could further facilitate the RISC and shorten emissive lifetimes (Fig. 1A), which may provide a springboard for faster scintillators. However, to the best of our knowledge, the effect of halogenation on radioluminescence (RL) properties is still unclear in TADF-based organic scintillators.

Following this principle, we designed halogen-modulated organic molecules with donor–acceptor architectures (Fig. 1B and Scheme S1). Halogen atoms (Br and I) are introduced to benefit the absorption of X-rays, while electron donor moiety of 9,9-dimethyl-9,10-dihydroacridine and electron acceptor moiety of 2,4-diphenyl-1,3,5-triazine are covalently linked to separate highest occupied molecular orbital (HOMO) and lowest unoccupied molecular orbital (LUMO), facilitating the decrease in Δ*E*_ST_ [41,52–54]. In such a manner, the generated triplet excitons following X-ray irradiation, approximately 75% of all excitons, can be harnessed through fast RISC to enhance RL. According to the strategy, we synthesized 10-(4-(4,6-diphenyl-1,3,5-triazin-2-yl)phenyl)-9,9-dimethyl-9,10-dihydroacridine (MAT) and its halogen derivatives 2,7-dibromo-10-(4-(4,6-diphenyl-1,3,5-triazin-2-yl)phenyl)-9,9-dimethyl-9,10-dihydroacridine (BMAT) and 2,7-diiodo-10-(4-(4,6-diphenyl-1,3,5-triazin-2-yl)phenyl)-9,9-dimethyl-9,10-dihydroacridine (Section S1). The chemical structures and purities of the 3 molecules were evaluated by nuclear magnetic resonance (NMR) spectroscopies (^1^H- and ^13^C-NMR) and high-performance liquid chromatography (HPLC) (Figs. S1 to S7).

## Results

### Photoluminescence behaviors of the molecules under ambient conditions

**Fig. 2.**
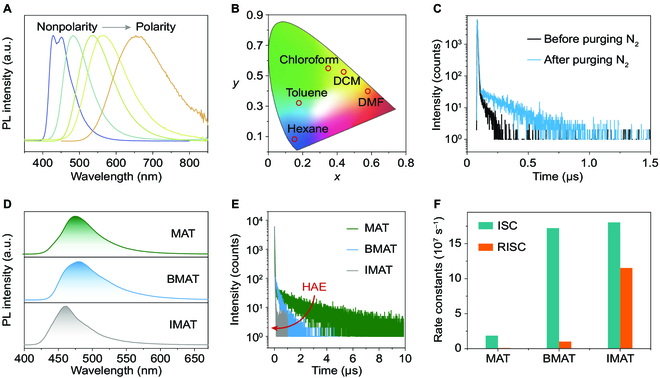
Photophysical properties of the TADF materials. (A) Normalized steady-state PL spectra of BMAT in different solutions (*c* = 1 × 10^−4^ M) under ambient conditions. From left to right, the solvents are hexane, toluene, chloroform, dichloromethane (DCM), and *N*,*N*-dimethylformamide (DMF), respectively. (B) CIE chromaticity coordinates variation of the PL colors in different solutions. (C) Transient PL decay profiles of BMAT in toluene solution before (black) and after (blue) purging nitrogen gas for 5 min. (D) Steady-state PL spectra of MAT, BMAT, and IMAT in the crystalline state, respectively. (E) Normalized transient PL decay curves of MAT, BMAT, and IMAT in the crystalline state, respectively. Upon halogenation, the heavy atom effect decreases the lifetimes. (F) Experimentally calculated rate constants of ISC (*k*_ISC_) and RISC (*k*_*R*ISC_) for the TADF materials. a.u., arbitrary units.

We first studied the photoluminescence (PL) behaviors of the molecules in solution under ambient conditions. All 3 molecules display positive solvatochromism, as depicted in Fig. 2A. For example, in nonpolar hexane, BMAT has a vibrational structure of PL band, which is ascribed to the locally excited state (^1^LE). After increasing polarity of solvents, the PL spectra of BMAT redshift gradually (Fig. 2B), accompanied by disappearance of the vibrational structure. The reason is that polar solvents feature stronger interaction of the solvent field with charge transfer (CT) excited state (larger dipole moment compared with LE state), making the energy level of ^1^CT lower than ^1^LE. In specific, the emission color of BMAT in *N*,*N*-dimethylformamide is located in the red region, which redshifts >200 nm than that in hexane. These results confirm the existence of a strong CT excited state in BMAT. It is worth noting that MAT and IMAT share similar properties (Fig. S8).

In a further set of experiments, we collected the emission spectra of the luminogens in dilute toluene solution at 77 K with and without delay, which can be utilized to calculate the energy level of the lowest singlet and triplet states. Accordingly, on the basis of the maxima emission peaks of the spectra, the Δ*E*_ST_ values of MAT, BMAT, and IMAT were estimated to be 22.7, 11.4, and 27.8 meV, respectively (Fig. S9). These energy barriers are small enough (<0.1 eV) to be overcome by thermal energy at room temperature, populating singlet excitons from the triplet excited states via RISC. We also recorded the transient PL decay curves under different conditions (air and nitrogen). As shown in Fig. 2C and Fig. S10, the lifetimes markedly increased after purging nitrogen to remove the dissolved O_2_ molecules, demonstrating the role of the triplet exciton for TADF emission of the 3 molecules. Furthermore, such decay curves match well with the double exponential decay model, which further proves the TADF nature of the molecules [35,46].

Subsequently, we explored the PL behaviors of the TADF molecules in solid state. Taking BMAT as an example, we demonstrated its aggregation-induced emission nature (Fig. S8), which is beneficial for the bright emission in solid state. As expected, all the 3 solid materials showed strong luminescence in the visible region, and the structureless emission spectra and large full width at half maximum demonstrate the CT nature of the lowest singlet state (S_1_) (Fig. 2D). Specifically, the total PL quantum yields (PLQYs) of MAT, BMAT, and IMAT were 98.3%, 22.2%, and 1.2%, respectively (Table S1). Within expectation, the introduction of heavy halogen atoms quenched the fluorescence, and the underlying photophysical processes deserve to be further investigated. To investigate the delayed fluorescence (DF) performance of these TADF materials, transient PL measurements were performed in solid state at room temperature (Fig. 2E). The prompt exciton lifetimes (*τ*_PF_) were determined as 16.8, 3.12, and 2.22 ns for MAT, BMAT, and IMAT, respectively. While the calculated delayed exciton lifetimes (*τ*_DF_) were 1.75, 0.225, and 0.0188 μs for MAT, BMAT, and IMAT, respectively (Table S1). With increasing the atomic number of halogen atoms, both prompt and delayed lifetimes showed a decreased tendency, which also follows the principle of the heavy atom effect. Utilizing exciton lifetimes and PLQY values, we calculated relative rate constants, including *k*_ISC_ and *k*_RISC_, which contribute to the light emission. As revealed in Fig. 2F and Table S1, BMAT and IMAT featured larger *k*_ISC_ and *k*_RISC_ values than MAT. However, the radiative rate from S_1_ to S_0_ ($k_{r}^{S}$) is small, and the nonradiative rate ($k_{\mathrm{nr}}^{S}$) of IMAT is too large (2.06 × 10^8^ s^−1^), resulting in its tiny PLQY.

To further study the properties of these TADF molecules, we performed time-dependent density function theory calculations. As shown in Fig. S11A to C, the natural transition orbitals of the lowest singlet excited state (S_1_) were localized on different segments, and the resultant small overlap further implies the CT nature of the S_1_ state. For frontier molecular orbitals (Fig. S12 and Table S2), their HOMO distributions were mainly localized on the 9,9-dimethyl-9,10-dihydroacridine unit, while their LUMO distributions were mainly located on the 2,4-diphenyl-1,3,5-triazine unit. Such well-separated characteristic between HOMOs and LUMOs generates small Δ*E*_ST_ (Fig. S11D to F), endowing themselves with TADF nature. Furthermore, the remarkably larger spin–orbit coupling coefficients (ξ) between S_1_ and T_2_ (Fig. S11D to F) indicate the participation of T_2_ state during ISC and RISC, which is consistent with the newly reported mechanisms of TADF [55].

### RL behaviors of the TADF molecules under ambient conditions

**Fig. 3.**
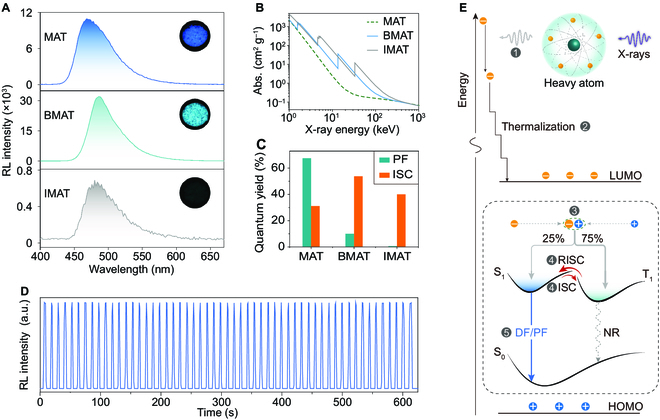
RL properties and our proposed mechanism of the TADF scintillators. (A) RL spectra of the crystalline TADF materials under X-rays with a dose rate of 278 μGy s^−1^. Insets are related photographs of the scintillators under exposure to X-ray. (B) The relationship between X-ray absorbances and X-ray energy of MAT (green dashed line), BMAT (blue solid line), and IMAT (gray solid line). The data were obtained from the photon cross-sectional database [58]. (C) Quantum yields of PF (*Φ*_PF_) and triplet excitons via ISC (*Φ*_ISC_). (D) The photostability of emission at 486 nm for BMAT crystals versus continuous on–off cycles of X-rays under a dose rate of 278 μGy s^−1^. (E) Proposed mechanism for RL in organic TADF scintillators. Orange cycles represent electrons and blue cycles are holes.

In the following experiments, we investigated the RL behaviors of these TADF materials in the crystalline state. From RL spectra (Fig. 3A), it can be observed that all the materials displayed the same emission wavelengths, compared with the related PL spectra. These results demonstrate the same ^1^CT characteristic of the lowest excited state following X-ray irradiation. As previously reported, the mechanisms between PL and RL in organic scintillators are different, with the latter one showing a larger proportion of triplet excitons. Similarly, for these TADF materials, their RL intensities showed varied tendency from PLQYs. In detail, BMAT displayed the strongest RL, while the RL of IMAT almost disappeared (Fig. 3A).

Aiming to explore the mechanism of the TADF scintillators deeply, we then compared the X-ray absorption abilities of the 3 materials (MAT: *Z*_max_ = 7, *K*_α_ = 0.392 keV; BMAT: *Z*_max_ = 35, *K*_α_ = 13.5 keV; IMAT: *Z*_max_ = 53, *K*_α_ = 33.2 keV) (Fig. 3B). The absorbances of X-ray are in the order of IMAT > BMAT > MAT, if we ignore the resonant absorption edges. Such tendency follows the rule of absorbing X-ray; that is, the attenuation coefficient is proportional to the 4th power of the atomic number *Z*.

Furthermore, we derived the formula of RL intensity, making it clear that the intensity is positively correlated with the *Φ*_PF_ and *Φ*_ISC_ (Eq. 11). Subsequently, we can explain the RL intensities of the molecules. Since the *Φ*_PF_ of IMAT is almost neglectable (*Φ*_PF_ = 0.0064; Fig. 3C and Table S1), its *Φ*_PF_ / (1 − *Φ*_ISC_) value is the smallest. While MAT and BMAT have a considerable value of either *Φ*_PF_ or *Φ*_ISC_, leading to a relatively larger *Φ*_PF_ / (1 − *Φ*_ISC_) value. From Eq. 11, we can also observe the positive role of absorbing X-rays. As a result, the polyoptimal X-ray absorbance and quantum yields empower BMAT with the most efficient RL among the 3 materials. Considering this, we conducted the properties, including photostability and detection limit, of BMAT under X-ray irradiation. Specifically, the emission intensity of the BMAT under the maximum dose of our equipment kept constant, even after 10 min at least (130 on–off circles; Fig. 3D). In addition, the detection limit was calculated to be 0.517 μGy s^−1^ (Fig. S13), which is lower by an order of magnitude than the dosage for X-ray medical diagnostics (5.5 μGy s^−1^) [56].

At last, we proposed a reasonable mechanism for metal-free organic scintillators based on TADF materials (Fig. 3E). First, X-ray photons mainly excite electrons (orange circles) from the inner shell of heavy atoms to generate high-energy holes (blue circles) (step 1). The generated fast photoelectrons subsequently induce abundant secondary electrons. Undergoing repeated process until ceasing ionization, electrons and holes thermalize in the LUMO and the HOMO, respectively (step 2). Subsequently, the thermalized electrons and holes recombine to produce singlet and triplet excitons in a ratio of 1:3 (step 3). Under reciprocating cycles of ISC and RISC (or vice versa; step 4), singlet excitons are finally populated to emit DF. On the other hand, the 25% singlet excitons in step 3 can directly emit prompt fluorescence (PF). Both the DF and PF contribute to the total RL (step 5).

### The application of radiography

**Fig. 4.**
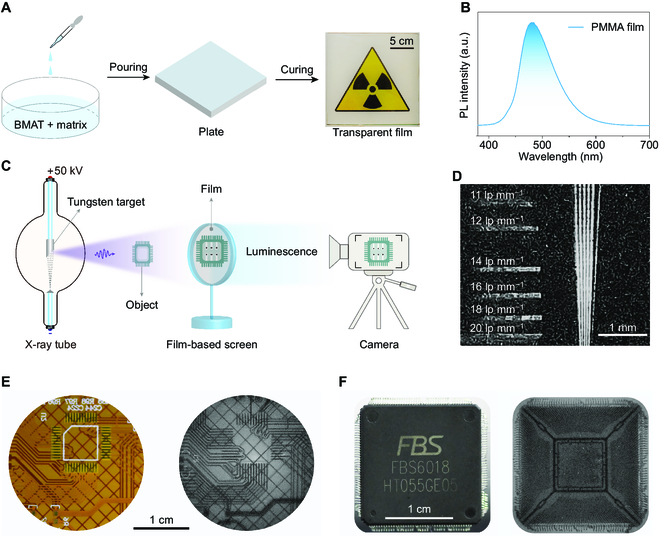
Illustration of the radiography application of the BMAT film. (A) Process for preparing large-area PMMA transparent films. The mass ratio between BMAT and matrixes was 0.5%. (B) Steady-state PL spectrum of the resulting transparent PMMA film. (C) Schematic of the setup for conducting X-ray contrast imaging. The object was placed between the X-ray tube and screen, while a camera with the remote controller was put from the opposite side of the screen. (D) An X-ray image of PMMA film for a standard X-ray test pattern plate. (E) Bright-field (left) and dark-field (right) photographs of a printed circuit board, recorded before and after X-ray irradiation, respectively. (F) Bright-field (left) and dark-field (right) photographs of a chip, recorded before and after X-ray irradiation, respectively. lp, line pairs.

Given the efficient scintillation performance of BMAT, we fabricated various transparent films to realize the application of radiography. Following the procedure in Fig. 4A, we prepared polymethyl methacrylate (PMMA) and polydimethylsiloxane films with BMAT, which all show high transparency (Fig. S15A to C). These films display similar emission spectra (Fig. 4B) and double exponential decay behaviors (Fig. S14), compared with BMAT crystals. Therefore, we employed a hand-held X-ray tube and digital camera to build an imaging system (Fig. 4C). Specifically, for the PMMA film, it showed a good imaging resolution with 20 line pairs mm^−1^ (Fig. 4D) seen by naked eyes. As a result, high-quality X-ray contrast imaging for printed circuit board and the chip was obtained successfully (Fig. 4E and F). It is worth noting that polydimethylsiloxane film did show good ability of X-ray imaging (Fig. S15D and E), demonstrating the potentially wide selection of matrix. This may empower the TADF scintillators to meet requirements in various scenes, for example, stretchable polymer matrixes should help to fibrate flexible X-ray detectors.

## Discussion

In conclusion, we have reported a TADF type of metal-free organic scintillators and provided a design concept for achieving efficient RL. These materials showed different luminescent behaviors under the excitation of X-ray and ultraviolet light. For example, although the PLQY of BMAT crystal is only 22.2%, which is lower than the 98.3% of MAT, BMAT featured the most intense RL with the assistance of strong X-ray absorption and efficient quantum yields of ISC. Through experimental and theoretical analyses, we figured out the importance of X-ray absorption and quantum yields of PF and ISC in TADF-based scintillators. From our findings, we also know that halogenated TADF emitters, especially the materials with iodine atoms, may suffer from lower quantum yield, which could impair the RL. Therefore, it is necessary to optimize molecular structures by modulating the parameters such as Δ*E*_ST_, oscillator strength, and the transition dipole moment. We believe that the halogenation of TADF emitters has bright prospects in organic scintillators. Our findings may provide a guideline for developing metal-free organic TADF scintillators, which will burden the type of organic scintillators to satisfy diverse application scenarios.

## Materials and Methods

### Measurements

^1^H and ^13^C NMR spectra were collected using a Bruker Ultra Shield Plus spectrometer (400 MHz). Chemical shifts were calibrated using tetramethylsilane in deuterated solvents as the internal standard. HPLC was performed using a SunFire C18 column conjugated to an ACQUITY UPLC H-class water HPLC system. Steady-state luminescence and excitation spectra were recorded using Hitachi F-7100 and Edinburgh FLS1000 fluorescence spectrophotometers. The lifetime was obtained on a fluorescence spectrophotometer (Edinburgh FLS1000) equipped with a xenon arc lamp (Xe900), a nanosecond hydrogen flash (nF920), or a microsecond flash (μF900). The luminescent photographs under the exposure of X-ray were taken by a Cannon EOS 700D camera equipped with a remote controller.

### Understanding the photophysical processes during TADF

In TADF, key parameters including the rate constants of ISC (*k*_ISC_), RISC (*k*_RISC_), and Δ*E*_ST_ are of importance. According to decay channels in Jablonski diagram, the decay rates of S_1_ and T_1_ after removing the excitation source can be written as:

$$\frac{d\left[S_{1}\right]}{d\left[t\right]}=-\left(k_{r}^{S}+k_{\mathrm{nr}}^{S}+k_{\mathrm{ISC}}\right)\left[S_{1}\right]+k_{\text{RISC}}\left[T_{1}\right]$$ (1)

$$\frac{d\left[T_{1}\right]}{d\left[t\right]}=-\left(k_{\mathrm{nr}}^{T}+k_{\text{RISC}}\right)\left[T_{1}\right]+k_{\mathrm{ISC}}\left[S_{1}\right]$$ (2)

where $k_{r}^{S}$, $k_{\mathrm{nr}}^{S}$, and *k*_ISC_ are rate constants of radiative decay, nonradiative decay, and ISC to triplet state from lowest singlet state (S_1_), respectively. $k_{\mathrm{nr}}^{T}$ and *k*_RISC_ are rate constants of nonradiative decay and RISC to singlet state from lowest triplet state (T_1_), respectively. Since TADF materials barely emit phosphorescence, the radiative rate constant of triplet state ($k_{r}^{T}$) can be assumed as 0.

By solving Eqs. 1 and 2, we can obtain:

$$\left[S_{1}\right]=A_{1}\exp\left[-k_{\mathrm{PF}}t\right]+A_{2}\exp\left[-k_{\mathrm{DF}}t\right]$$ (3)

where *A*_1_ and *A*_2_ are the amplitudes and *k*_PF_ and *k*_DF_ are the rate constants of PF and DF, respectively.

Specifically, the *k*_PF_ and *k*_DF_ can be given as:

$$\begin{array}{c} k_{\mathrm{PF}},k_{\mathrm{DF}}=\frac{k_{r}^{S}+k_{\mathrm{nr}}^{S}+k_{\mathrm{ISC}}+k_{\mathrm{nr}}^{T}+k_{\text{RISC}}}{2}\\ \;\times \left(1\pm \sqrt{1-\frac{4\left(k_{r}^{S}+k_{\mathrm{nr}}^{S}+k_{\mathrm{ISC}}\right)\left(k_{\mathrm{nr}}^{T}+k_{\text{RISC}}\right)-4k_{\mathrm{ISC}}k_{\text{RISC}}}{{\left(k_{r}^{S}+k_{\mathrm{nr}}^{S}+k_{\mathrm{ISC}}+k_{\mathrm{nr}}^{T}+k_{\text{RISC}}\right)}^{2}}}\right) \end{array}$$ (4)

The experimentally measurable total PLQYs (*Φ*_PL_) is the sum of prompt (*Φ*_PF_) and delayed (*Φ*_DF_) components. In addition, *Φ*_DF_ can be expressed as:

$$\Phi_{\mathrm{DF}}=\sum_{m=1}^{\infty }\Phi_{\mathrm{PF}}{\left(\Phi_{\mathrm{ISC}}\Phi_{\text{RISC}}\right)}^{m}=\frac{\Phi_{\mathrm{ISC}}\Phi_{\text{RISC}}}{1-\Phi_{\mathrm{ISC}}\Phi_{\text{RISC}}}\Phi_{\mathrm{PF}}$$ (5)

$$\text{where}\;\Phi_{\mathrm{PF}}=\frac{k_{r}^{S}}{k_{r}^{S}+k_{\mathrm{nr}}^{S}+k_{\mathrm{ISC}}}$$ (6)

Under reasonable assumptions of $k_{r}^{T}$ = 0 and $k_{\mathrm{nr}}^{T}$ ≈ 0, it can be obtained that (for details, see Ref. [46])

$$k_{\text{RISC}}\approx \frac{k_{\mathrm{PF}}+k_{\mathrm{DF}}}{2}-\sqrt{{\left(\frac{k_{\mathrm{PF}}+k_{\mathrm{DF}}}{2}\right)}^{2}-k_{\mathrm{PF}}k_{\mathrm{DF}}\left(1+\frac{\Phi_{\mathrm{DF}}}{\Phi_{\mathrm{PF}}}\right)}$$ (7)

$$k_{\mathrm{ISC}}=\frac{k_{\mathrm{PF}}k_{\mathrm{DF}}}{k_{\text{RISC}}}\frac{\Phi_{\mathrm{DF}}}{\Phi_{\mathrm{PF}}}$$ (8)

$$k_{r}^{S}=\frac{k_{\mathrm{PF}}k_{\mathrm{DF}}}{k_{\text{RISC}}}\Phi_{\mathrm{PL}}$$ (9)

$$k_{\mathrm{nr}}^{S}=\frac{k_{\mathrm{PF}}k_{\mathrm{DF}}}{k_{\text{RISC}}}\left(1-\Phi_{\mathrm{PL}}\right)$$ (10)

In Eqs. 7 to 10, the physical parameters such as *k*_PF_, *k*_DF_, *Φ*_PF_, and *Φ*_DF_ can be obtained from the experimental PLQYs and transient decay profiles, using emission lifetime (*τ*_PF_, *τ*_DF_) and fitting parameter (*A*_1_, *A*_2_) [57].

As the case of TADF-based organic scintillators, we can derive the formula of RL intensity:

$$\begin{array}{c} \begin{array}{cc} I_{\mathrm{RL}} & \;=\mathrm{Abs}.\times \delta \times \eta \times \left\{0.25\Phi_{\mathrm{PF}}+\sum_{k=0}^{\infty }0.75\Phi_{\mathrm{PF}}\Phi_{\text{RISC}}{\left(\Phi_{\mathrm{ISC}}\Phi_{\text{RISC}}\right)}^{k}+\sum_{k=1}^{\infty }0.25\Phi_{\mathrm{PF}}{\left(\Phi_{\mathrm{ISC}}\Phi_{\text{RISC}}\right)}^{k}\right\}\\ & \;=\frac{\rho Z^{4}}{{\mathrm{AE}}^{3}}\times \delta \times \eta \times \left\{0.25\Phi_{\mathrm{PF}}+\sum_{k=0}^{\infty }0.75\Phi_{\mathrm{PF}}\Phi_{\text{RISC}}{\left(\Phi_{\mathrm{ISC}}\Phi_{\text{RISC}}\right)}^{k}+\sum_{k=1}^{\infty }0.25\Phi_{\mathrm{PF}}{\left(\Phi_{\mathrm{ISC}}\Phi_{\text{RISC}}\right)}^{k}\right\}\\ & \;=\frac{\rho Z^{4}}{{\mathrm{AE}}^{3}}\times \delta \times \eta \times 0.25\times \Phi_{\mathrm{PF}}\times \left\{1+\frac{3\Phi_{\text{RISC}}}{1-\Phi_{\mathrm{ISC}}\Phi_{\text{RISC}}}+\frac{\Phi_{\mathrm{ISC}}\Phi_{\text{RISC}}}{1-\Phi_{\mathrm{ISC}}\Phi_{\text{RISC}}}\right\}\\ & \;\approx \frac{\rho Z^{4}}{{\mathrm{AE}}^{3}}\times \delta \times \eta \times \Phi_{\mathrm{PF}}\times \frac{1}{1-\Phi_{\mathrm{ISC}}} \end{array} \end{array}$$ (11)

where *ρ* is the material density, *Z* is the atomic number, *A* is the atomic mass, *E* is the X-ray photon energy, *δ* is the pairs of electron hole generated by one X-ray photon, and *η* is the efficiency in carrier transport process.

From Eq. 11, we can figure out the RL intensity within TADF-based scintillators. That is, the RL intensity (*I*_RL_) displays positive relationship with atomic number *Z*, PF quantum yield (*Φ*_PF_), and quantum yield of ISC (*Φ*_ISC_). We can explain the RL tendency of the scintillators, by analyzing the factors of *Φ*_PF_ and *Φ*_ISC_.

## Data Availability

All of the relevant data that support the findings in this work are available upon request from the corresponding author under reasonable request.

## Supplementary Materials

Supplementary 1 Section S1. Experimental section. Section S2. Understanding the photophysical processes during TADF. Section S3. Behaviors of the TADF molecules in dilute solutions. Section S4. Density function theory calculation. Section S5. Detection limit of BMAT scintillator. Section S6. Properties of BMAT transparent films. Scheme S1. Molecular structures of the TADF molecules and their synthetic routes. Fig. S1. The ^1^H NMR spectrum of MAT molecule in CDCl_3_. Fig. S2. The ^13^C NMR spectrum of MAT molecule in CDCl_3_. Fig. S3. The ^1^H NMR spectrum of BMAT molecule in CDCl_3_. Fig. S4. The ^13^C NMR spectrum of BMAT molecule in CDCl_3_. Fig. S5. The ^1^H NMR spectrum of IMAT molecule in CDCl_3_. Fig. S6. The ^13^C NMR spectrum of IMAT molecule in CDCl_3_. Fig. S7. HPLC chromatogram of TADF molecule in methanol. Fig. S8. Photophysical properties in different dilute solutions under excitation of ultraviolet light. Fig. S9. Steady-state and delayed emission spectra of the scintillators. Fig. S10. Room-temperature transient PL decay profiles. Fig. S11. Electronic configurations and energy level diagrams of the materials. Fig. S12. Theoretical calculation for TADF behaviors of the scintillators. Fig. S13. Evaluating the detection limit of BMAT. Fig. S14. Transient PL decay curves of BMAT transparent films. Fig. S15. Radiography application of BMAT-based films. Table S1. Photophysical parameters of the TADF molecules. Table S2. Molecular orbitals and related proportions of the scintillators based on S_1_.
