# Supplementary material for: Halogenated Thermally Activated Delayed Fluorescence Materials for Efficient Scintillation
Source: Research (Wash D C). 2023 Mar 27;6:0090. doi: 10.34133/research.0090 (PMC10044329; doi:10.34133/research.0090)
Supplement: Supplementary 1 — Section S1. Experimental section. Section S2. Understanding the photophysical processes during TADF. Section S3. Behaviors of the TADF molecules in dilute solutions. Section S4. Density function theory calculation. Section S5. Detection limit of BMAT scintillator. Section S6. Properties of BMAT transparent films. Scheme S1. Molecular structures of the TADF molecules and their synthetic routes. Fig. S1. The 1H NMR spectrum of MAT molecule in CDCl3. Fig. S2. The 13C NMR spectrum of MAT molecule in CDCl3. Fig. S3. The 1H NMR spectrum of BMAT molecule in CDCl3. Fig. S4. The 13C NMR spectrum of BMAT molecule in CDCl3. Fig. S5. The 1H NMR spectrum of IMAT molecule in CDCl3. Fig. S6. The 13C NMR spectrum of IMAT molecule in CDCl3. Fig. S7. HPLC chromatogram of TADF molecule in methanol. Fig. S8. Photophysical properties in different dilute solutions under excitation of ultraviolet light. Fig. S9. Steady-state and delayed emission spectra of the scintillators. Fig. S10. Room-temperature transient PL decay profiles. Fig. S11. Electronic configurations and energy level diagrams of the materials. Fig. S12. Theoretical calculation for TADF behaviors of the scintillators. Fig. S13. Evaluating the detection limit of BMAT. Fig. S14. Transient PL decay curves of BMAT transparent films. Fig. S15. Radiography application of BMAT-based films. Table S1. Photophysical parameters of the TADF molecules. Table S2. Molecular orbitals and related proportions of the scintillators based on S1. [file research.0090.f1.docx]

**Supporting Information**

**Halogenated Thermally Activated Delayed Fluorescence Materials for Efficient Scintillation**

Xiao Wang^1,4†^, Guowei Niu^2†^, Zixing Zhou^1^, Zhicheng Song^1^, Ke Qin^2^, Xiaokang Yao^2^, Zhijian Yang^3^, Xiaoze Wang^3^, He Wang^2^, Zhuang Liu^2^, Chengzhu Yin^2^, Huili Ma^2^, Kang Shen^2^, Huifang Shi^2,5^, Jun Yin^6^, Qiushui Chen^3*^, Zhongfu An^1,2*^ and Wei Huang^1,2,4,5*^

^1^The Institute of Flexible Electronics (IFE, Future Technologies), Xiamen University, Xiamen, 361005, China.

^2^Key Laboratory of Flexible Electronics (KLOFE) & Institute of Advanced Materials (IAM), Nanjing Tech University, Nanjing, 211816, China.

^3^MOE Key Laboratory for Analytical Science of Food Safety and Biology, State Key Laboratory of Photocatalysis on Energy and Environment, College of Chemistry, Fuzhou University, Fuzhou, 350108, China.

^4^Frontiers Science Center for Flexible Electronics (FSCFE), MIIT Key Laboratory of Flexible Electronics (KLoFE), Northwestern Polytechnical University, Xi'an 710072, China.

^5^State Key Laboratory of Organic Electronics and Information Displays & Institute of Advanced Materials (IAM), Nanjing University of Posts & Telecommunications, 9 Wenyuan Road, Nanjing 210023, China.

^6^Department of Applied Physics, The Hong Kong Polytechnic University, Kowloon 999077 Hong Kong, China.

*Correspondence should be addressed to Qiushui Chen; [qchen@fzu.edu.cn,](mailto:qchen@fzu.edu.cn,) Zhongfu An; [iamzfan@njtech.edu.cn](mailto:iamzfan@njtech.edu.cn) and Wei Huang; vc@nwpu.edu.cn

†These authors contributed equally to this work.

**Contents**

[**I. Experimental section. S2**](#_Toc93198242)

[**II. Understanding the photophysical processes during TADF. S7**](#_Toc93198243)

[**III. Behaviors of the TADF molecules in dilute solutions. S7**](#_Toc93198244)

[**IV. Density function theory (DFT) calculation. S9**](#_Toc93198245)

[**V. Detection limit of BMAT scintillator. S11**](#_Toc93198246)

[**VI. Properties of BMAT transparent films. S11**](#_Toc93198247)

[**VII. References S12**](#_Toc93198248)

# I. Experimental section

**General.** Unless specially mentioned, all the raw reagents and organic solvents were obtained from chemical sources and directly used. The related organic reactions were proceeded under nitrogen atmosphere using classical Schlenk technology, if we do not provide another description. Before using, tetrahydrofuran (THF) was dried and distilled with sodium (Na) and benzophenone added as color indicators. All products were purified *via* flash column chromatography methodology, with original analysis by thin-layer chromatography (TLC) plates. After flash column chromatography, we further utilized crystallization techniques such as slow evaporation or slow cooling to obtain the crystals. The solvents for investigating photophysical properties satisfy the quality of chromatographic grade. The absolute photoluminescence quantum yields (PLQYs) of the materials were determined using UV-NIR absolute PL quantum yield spectrometer C13534-11. Specifically, the PLQYs of materials are defined as the value of number of photons emitted as photoluminescence divided by number of photons absorbed by samples. The spectrometer provides simultaneously the absorption and photoluminescence spectra of one sample, so that we can integrate both of them and obtain the PLQYs.


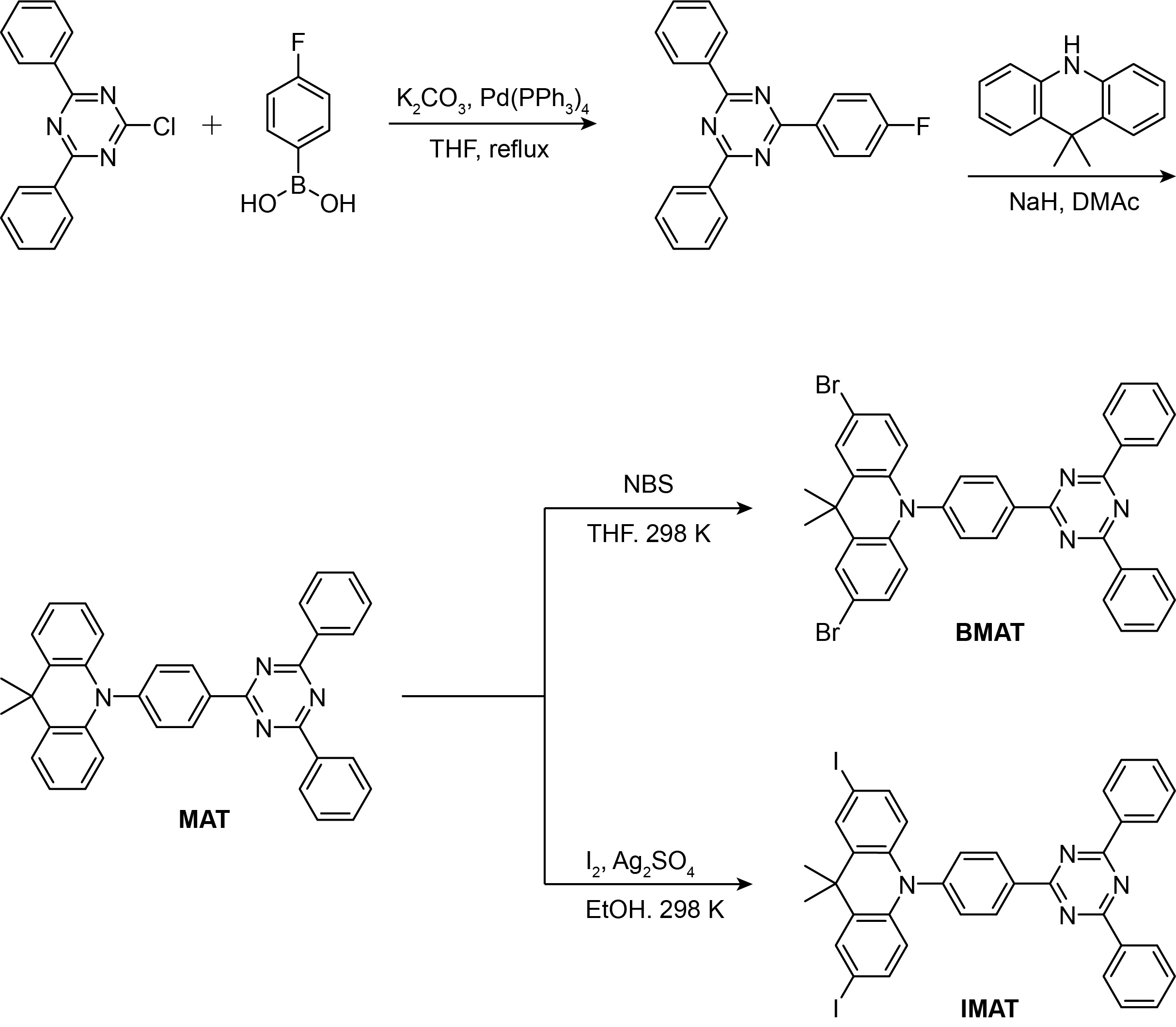


**Scheme S1. Molecular structures of the TADF molecules and their synthetic routes.**

**Synthesis of 2-(4-fluorophenyl)-4,6-diphenyl-1,3,5-triazine (FPT)**. This molecule was prepared by a Suzuki coupling reaction. Specifically, in a two-necked flask with a condenser, (4-fluorophenyl)boronic acid (5 g, 35.7 mmol), 2-chloro-4,6-diphenyl-1,3,5-triazine (6.4 g, 23.8 mmol), Tetrakis(triphenylphosphine)palladium (0.55 g, 0.48 mmol), and potassium carbonate (6.6 g, 47.6 mmol) were added. After providing a nitrogen atmosphere, 50 mL THF and 10 mL D.I. water were slowly injected. Subsequently, the resultant mixture was stirred at 353 K for 12 hours. Once the reaction completed, the crude solution was evaporated and extracted with dichloromethane for three times. The obtained organic layer was dried using anhydrous sodium sulfate (Na_2_SO_4_). After dichloromethane was further removed, the residue was purified by flash column chromatography to give FPT (6.3 g, 81%) as a white needle-like crystal. ^1^H NMR (CDCl_3_): δ 8.85-8.73 (m, 6H), 7.67-7.55 (m, 6H), 7.31-7.26 (m, 2H), which is consistent with previously reported data (Ref. S1).

**Synthesis of 10-(4-(4,6-diphenyl-1,3,5-triazin-2-yl)phenyl)-9,9-dimethyl-9,10-dihydroacridine (MAT)**. In a two-necked nitrogen-filled flask, 9,9-dimethyl-9,10-dihydroacridine (1.0 g, 4.6 mmol) and sodium hydride (60% dispersion in mineral oil, 0.37 g, 9.2 mmol) were added. After which, 25 mL dry dimethylacetamide (DMAc) was injected by syringe at 273 K, the solution was stirred for 1 hour. Then the solution was added dropwise, at 273 K, into another nitrogen-filled flask charged with FPT (1.5 g, 4.6 mmol) and 5 mL dry DMAc, the mixture was stirred at 373 K for 12 hours. The reaction was quenched by adding water, accompanied with evaporation to remove solvents. Then the solids were extracted with dichloromethane for three times. The resulting crude product was purified by flash column chromatography to give MAT (1.4 g, 60%) as a pale-yellow solid. ^1^H NMR (CDCl_3_): δ 9.08-9.01 (m, 2H), 8.90-8.78 (m, 4H), 7.70-7.55 (m, 8H), 7.51 (dd, *J* = 7.5, 1.8 Hz, 2H), 7.06-6.93 (m, 4H), 6.40 (dd, *J* = 7.9, 1.5 Hz, 2H), 1.75 (s, 6H). ^13^C NMR (CDCl_3_): δ 171.92, 171.15, 145.38, 140.69, 136.19, 136.14, 132.84, 131.73, 131.64, 130.30, 129.13, 128.84, 126.58, 125.49, 120.96, 114.28, 36.16, 31.44. The results are consistent with previously reported data (Ref. S2).

**Synthesis of 2,7-dibromo-10-(4-(4,6-diphenyl-1,3,5-triazin-2-yl)phenyl)-9,9-dimethyl-9,10-dihydroacridine (BMAT)**. In a two-necked nitrogen-filled flask, MAT (0.5 g, 0.97 mmol) and 10 mL dry THF were added. In another nitrogen-filled flask, N-bromosuccinimide (NBS, 0.38 g, 2.1 mmol) and 10 mL dry THF were mixed. After which, the solution of NBS was added dropwise into the solution of MAT at 273 K, and the mixture was stirred at 298 K for 12 hours. When the reaction completed, evaporation was given to remove organic solvent. Then the solids were extracted with dichloromethane for three times. The resulting crude product was purified by flash column chromatography to give BMAT (0.5 g, 80%) as a pale-yellow solid. ^1^H NMR (CDCl_3_): δ 9.06-9.00 (m, 2H), 8.85-8.77 (m, 4H), 7.69-7.58 (m, 6H), 7.55 (d, *J* = 2.3 Hz, 2H), 7.53-7.49 (m, 2H), 7.09 (dd, *J* = 8.8, 2.3 Hz, 2H), 6.24 (d, *J* = 8.8 Hz, 2H), 1.69 (s, 6H). ^13^C NMR (CDCl_3_): δ 171.98, 170.93, 144.35, 139.53, 136.79, 136.02, 132.92, 131.95, 131.83, 131.27, 129.51, 129.12, 128.86, 128.31, 115.98, 113.72, 36.44, 31.11. The results are consistent with previously reported data (Ref. S3).

**Synthesis of 10-(4-(4,6-diphenyl-1,3,5-triazin-2-yl)phenyl)-2,7-diiodo-9,9-dimethyl-9,10-dihydroacridine (IMAT)**. In a round flask, MAT (0.2 g, 0.39 mmol), iodine (0.33 g, 1.3 mmol), silver sulfate (0.36 g, 1.2 mmol), and 20 mL EtOH were mixed under ambient conditions. The mixture was stirred overnight at 298 K. When the reaction completed, it was washed with sodium thiosulfate (0.1 M) and the residue after evaporated was purified by column chromatography to provide IMAT (0.2 g, 67%) as a pale-yellow solid (3.10 g, 81%). ^1^H NMR (CDCl_3_): δ 9.05-9.01 (m, 2H), 8.84-8.79 (m, 4H), 7.70 (d, *J* = 2.0 Hz, 2H), 7.68-7.57 (m, 6H), 7.52-7.47 (m, 2H), 7.27 (dd, *J* = 7.8, 2.1 Hz, 2H), 6.11 (d, *J* = 8.7 Hz, 2H), 1.67 (s, 6H). ^13^C NMR (CDCl_3_): δ 171.99, 170.92, 144.15, 140.13, 136.83, 136.03, 135.45, 134.20, 132.90, 132.45, 131.94, 131.21, 129.12, 128.85, 116.52, 83.70, 36.09, 31.26.


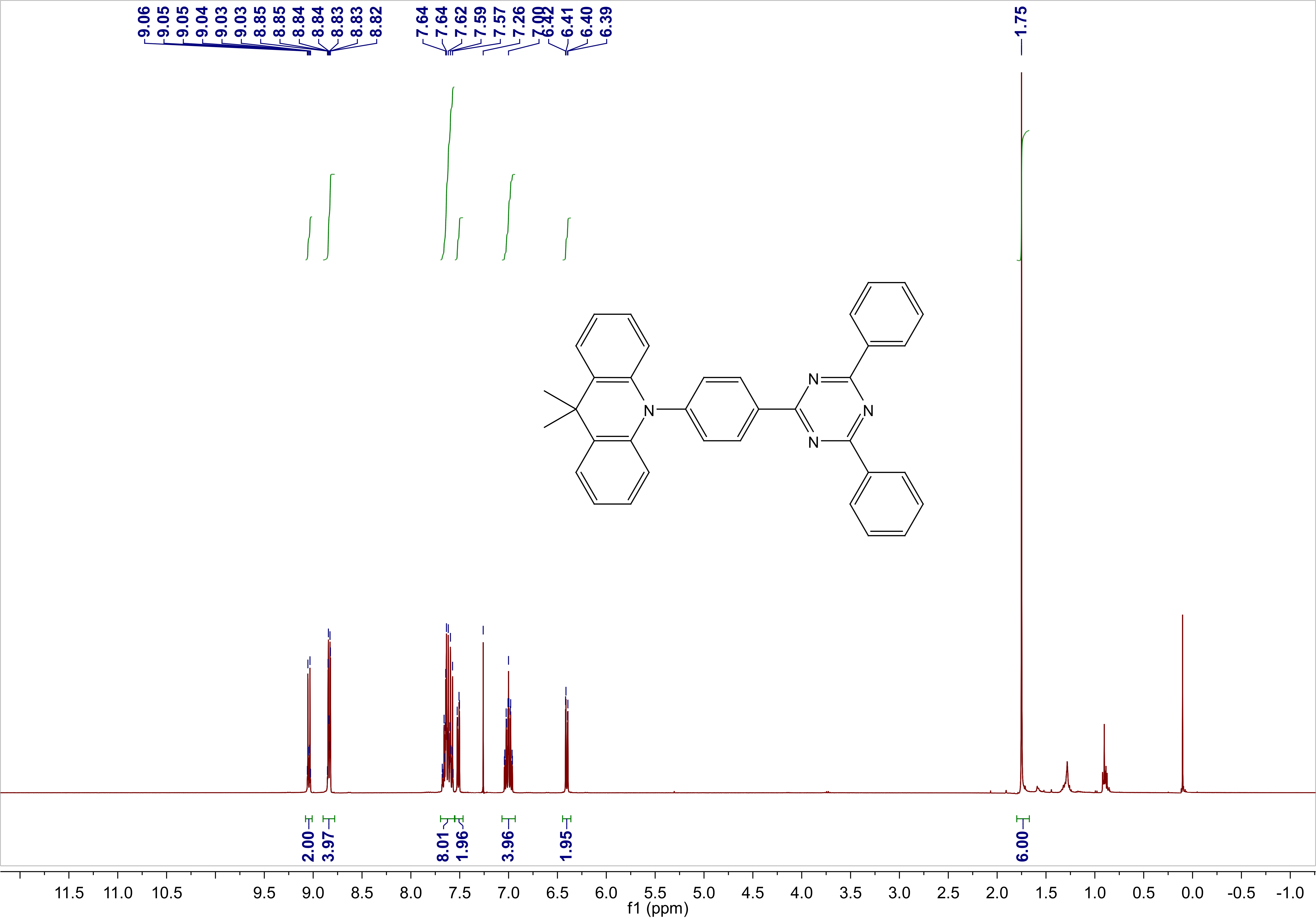


**Figure S1. The ^1^H NMR spectrum of MAT molecule in CDCl_3_.**


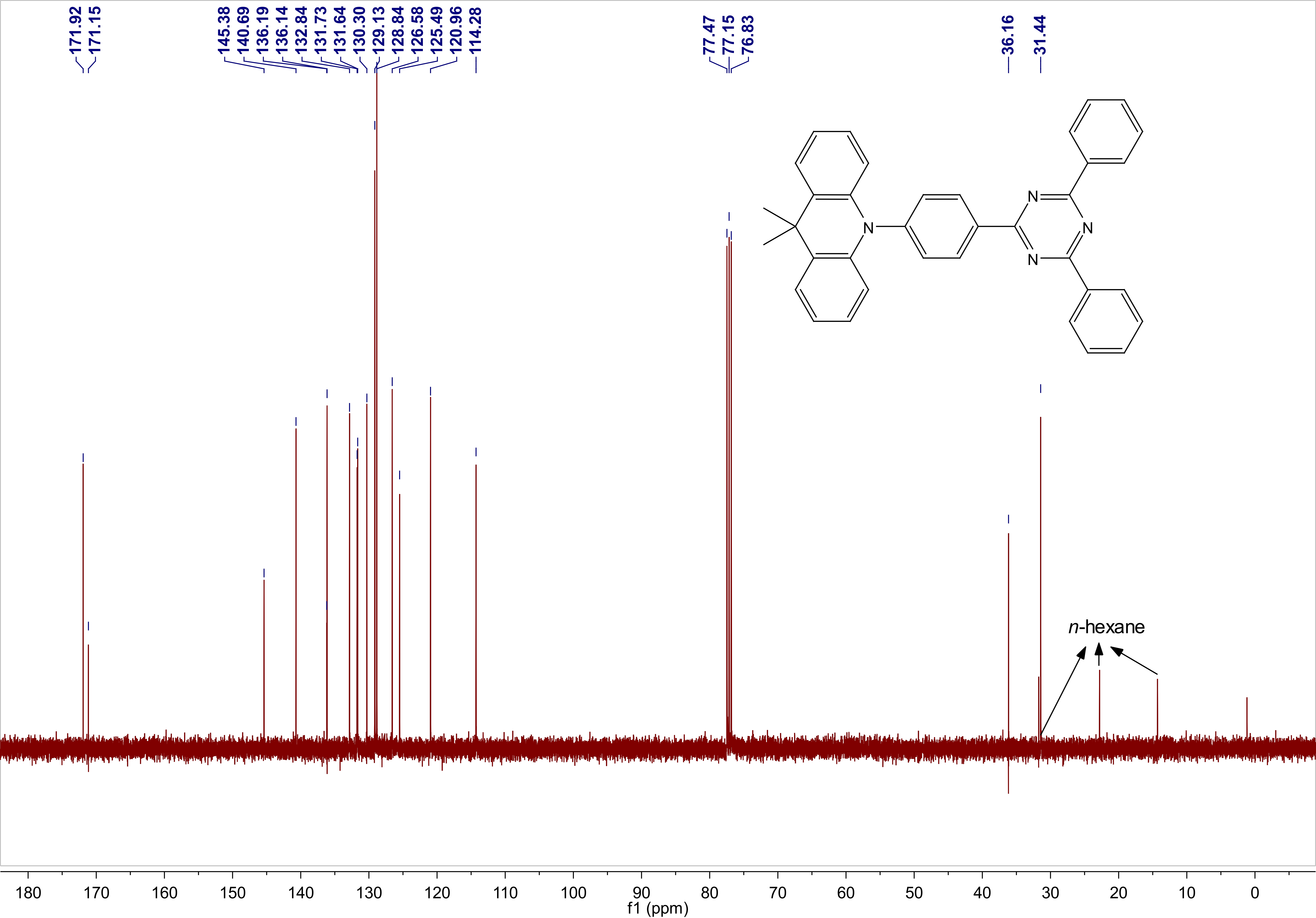


**Figure S2. The ^13^C NMR spectrum of MAT molecule in CDCl_3_.**


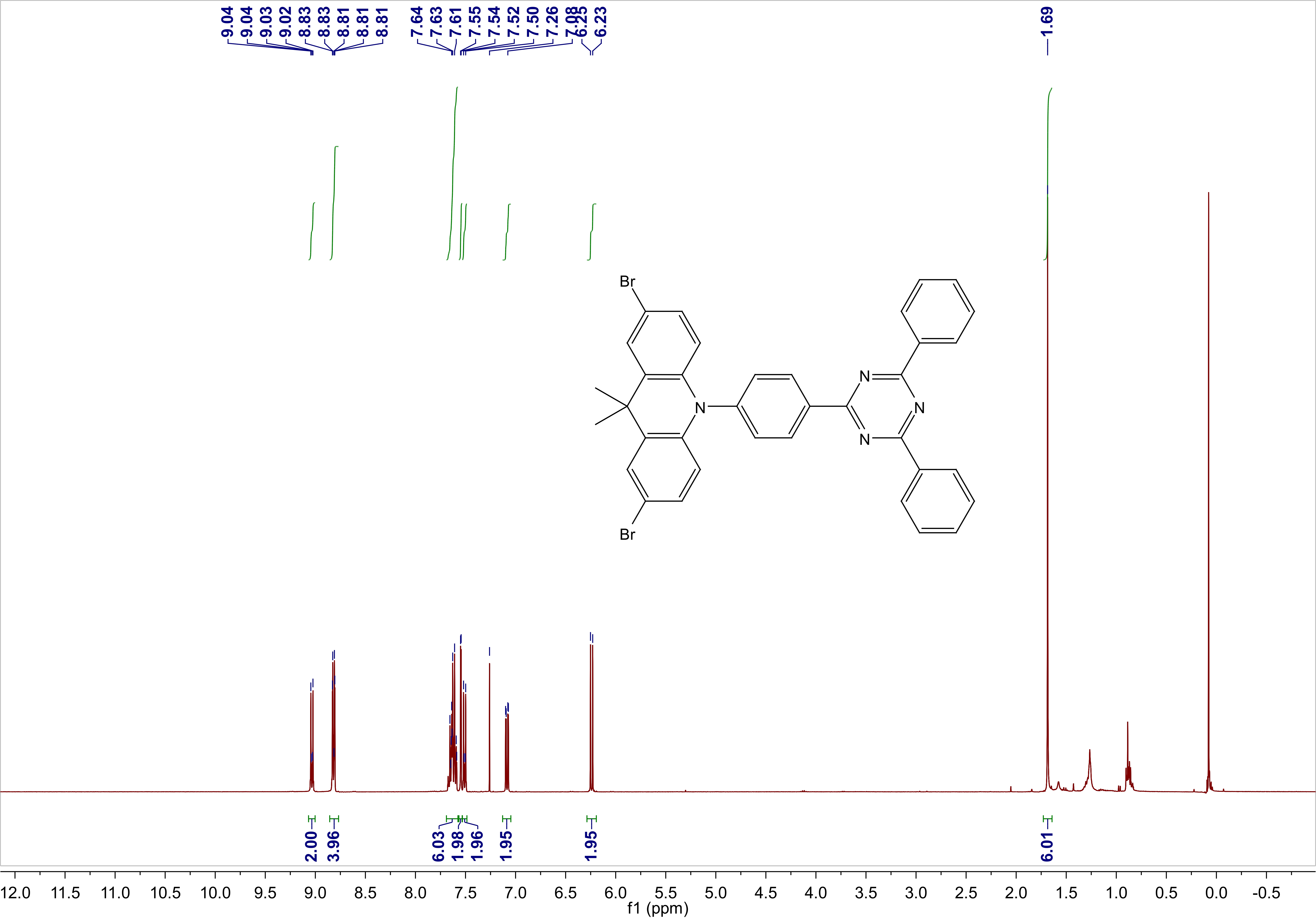


**Figure S3. The ^1^H NMR spectrum of BMAT molecule in CDCl_3_.**


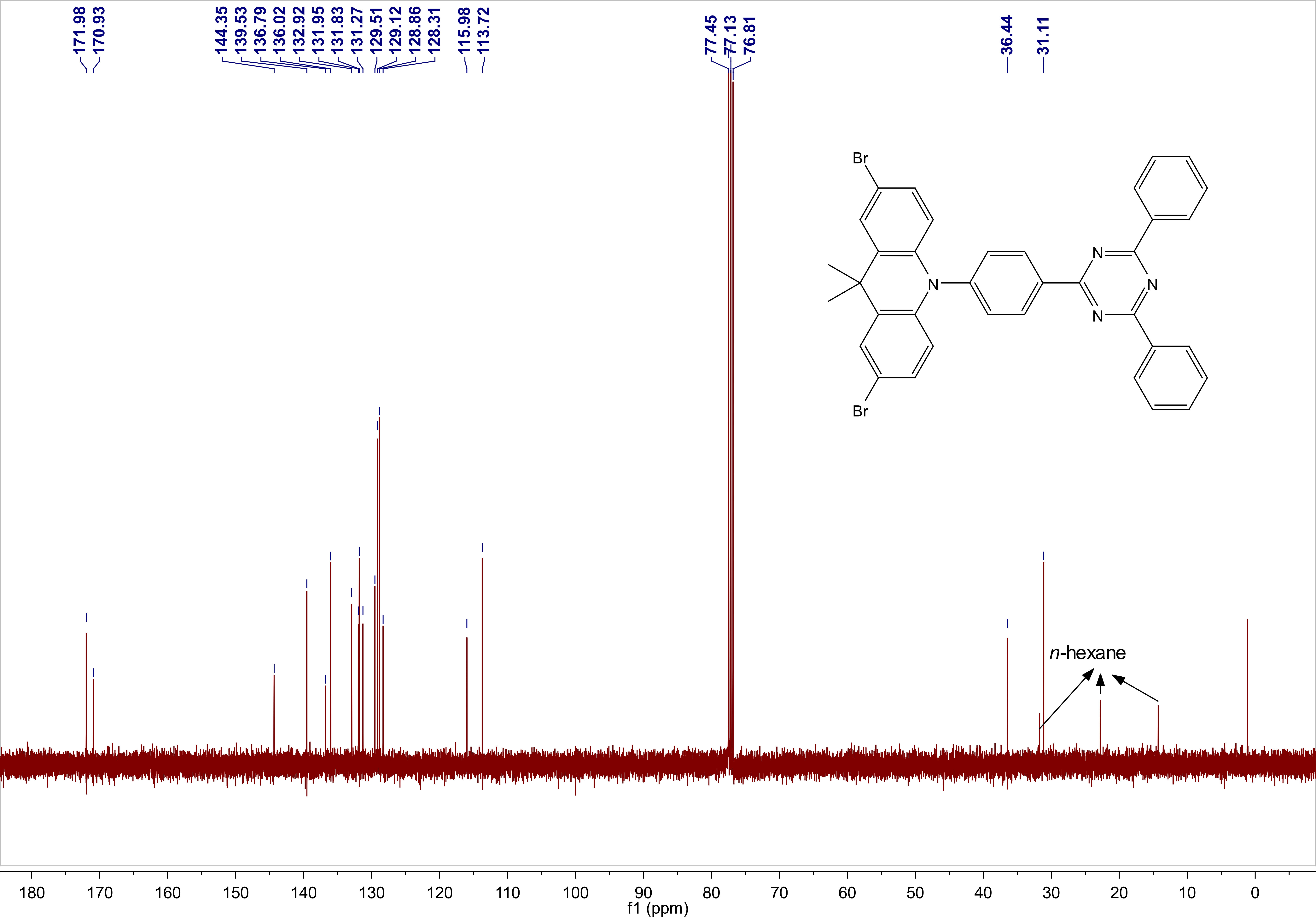


**Figure S4. The ^13^C NMR spectrum of BMAT molecule in CDCl_3_.**


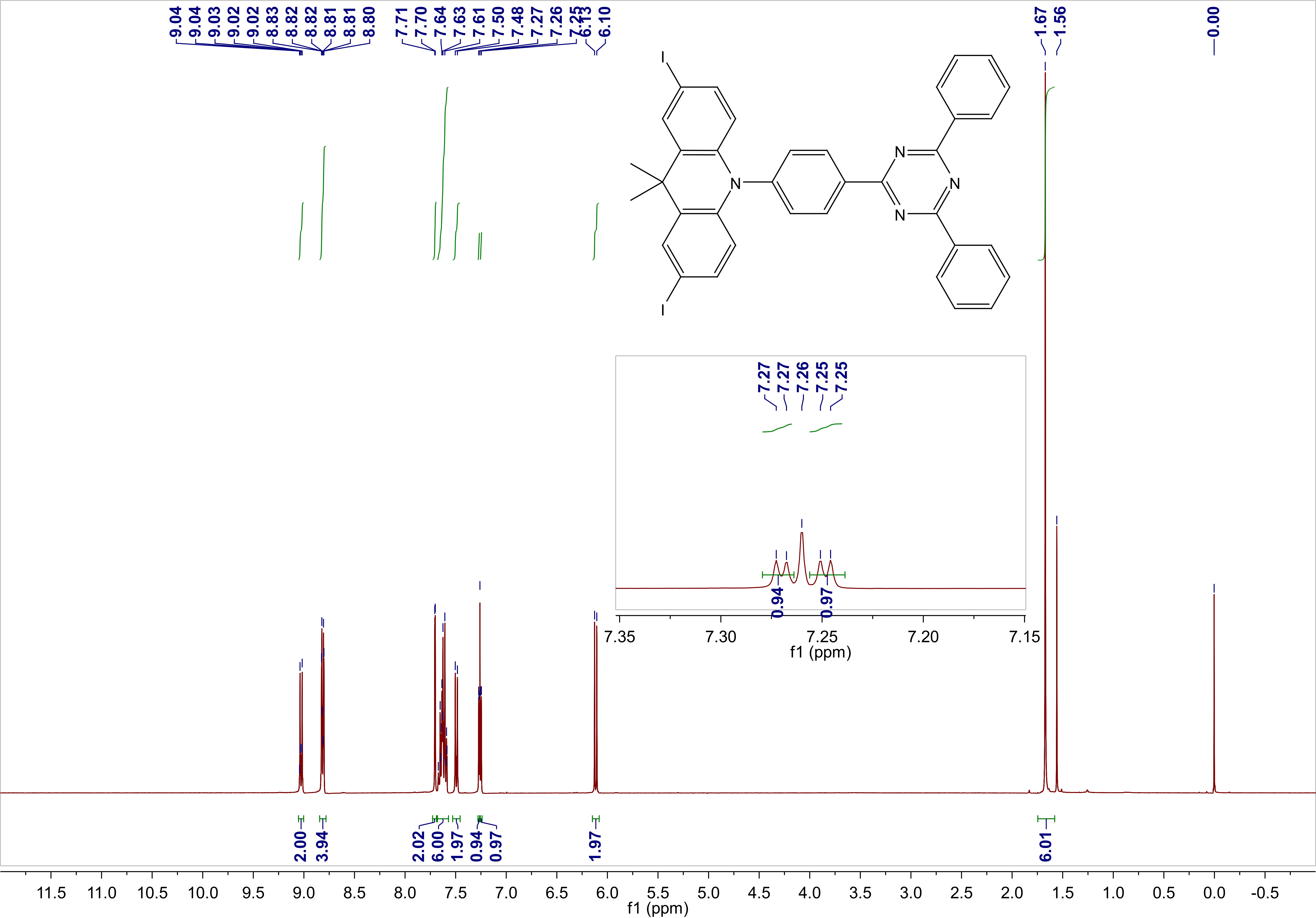


**Figure S5. The ^1^H NMR spectrum of IMAT molecule in CDCl_3_.**


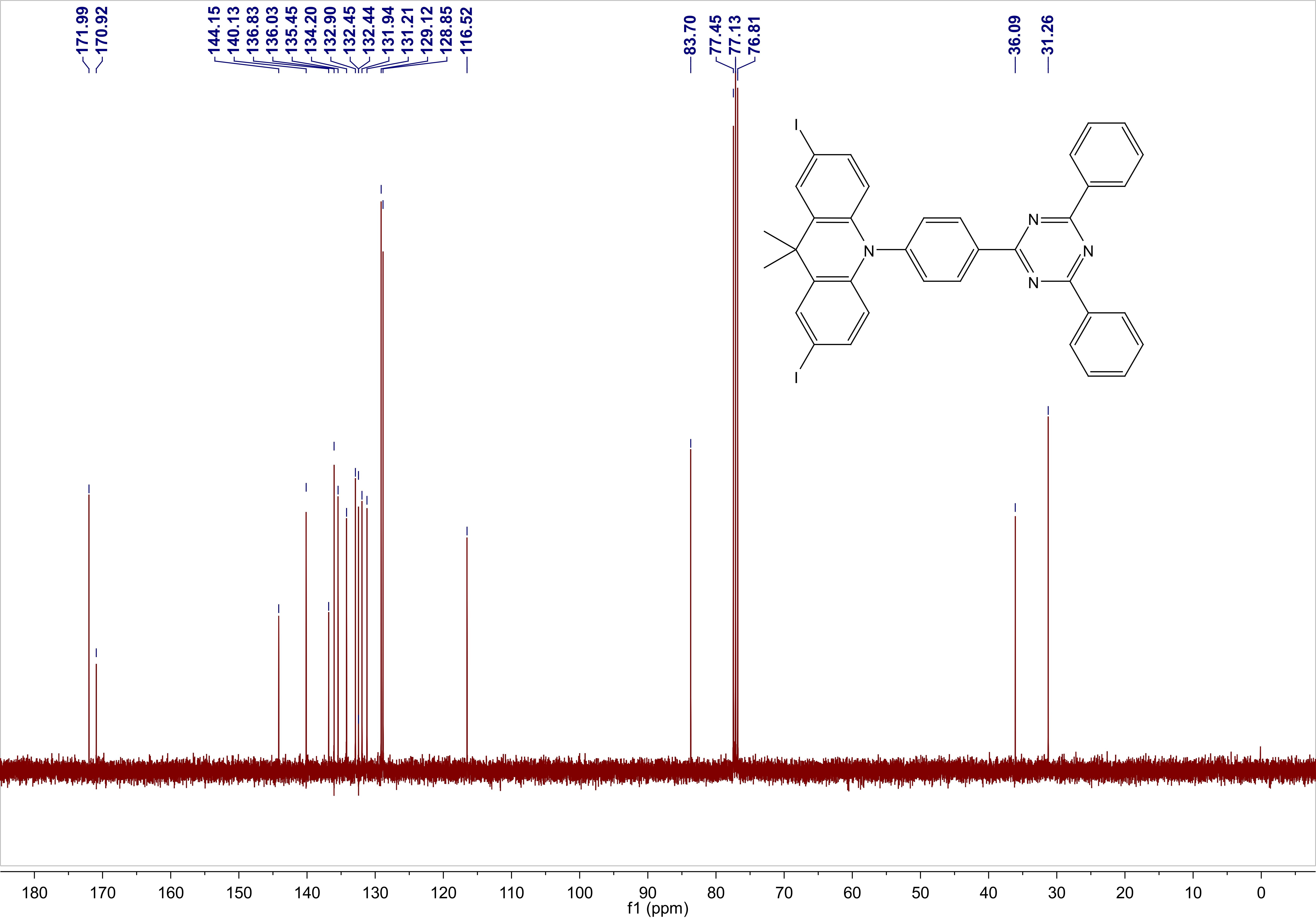


**Figure S6. The ^13^C NMR spectrum of IMAT molecule in CDCl_3_.**


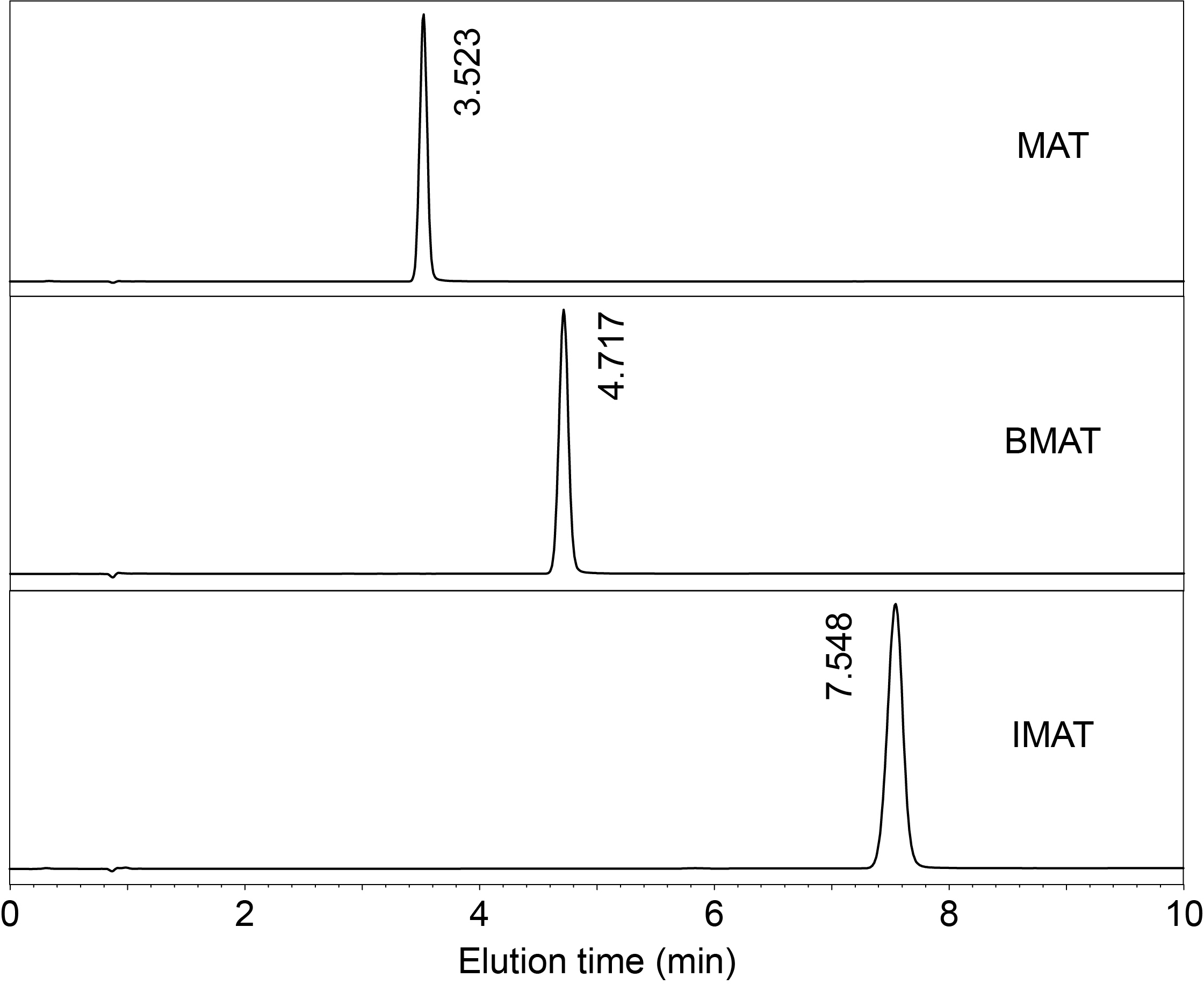


**Figure S7. High-performance liquid chromatography chromatogram of TADF molecule in methanol.**

# II. Understanding the photophysical processes during TADF

**Table S1.** **Photophysical parameters of the TADF molecules.**

| Compd. | *Ф*_PL_ | *Ф*_PF_ | *Ф*_DF_ | *τ*_PF_ (s) | *τ*_DF_ (s) | *k*_ISC_ (s^-1^) | *k*_RISC_ (s^-1^) | *kS r* (s^-1^) | *kS nr* (s^-1^) | *Ф*_ISC_ |
| --- | --- | --- | --- | --- | --- | --- | --- | --- | --- | --- |
| MAT | 0.983 | 0.675 | 0.308 | 1.68×10^-8^ | 1.75×10^-6^ | 1.86×10^7^ | 8.34×10^5^ | 4.00×10^7^ | 6.93×10^5^ | 0.312 |
| BMAT | 0.222 | 0.100 | 0.122 | 3.12×10^-9^ | 2.25×10^-7^ | 1.72×10^8^ | 9.99×10^6^ | 3.16×10^7^ | 1.11×10^8^ | 0.538 |
| IMAT | 0.012 | 0.0064 | 0.0056 | 2.22×10^-9^ | 1.88×10^-8^ | 1.80×10^8^ | 1.15×10^8^ | 2.50×10^6^ | 2.06×10^8^ | 0.400 |

# III. Behaviors of the TADF molecules in dilute solutions


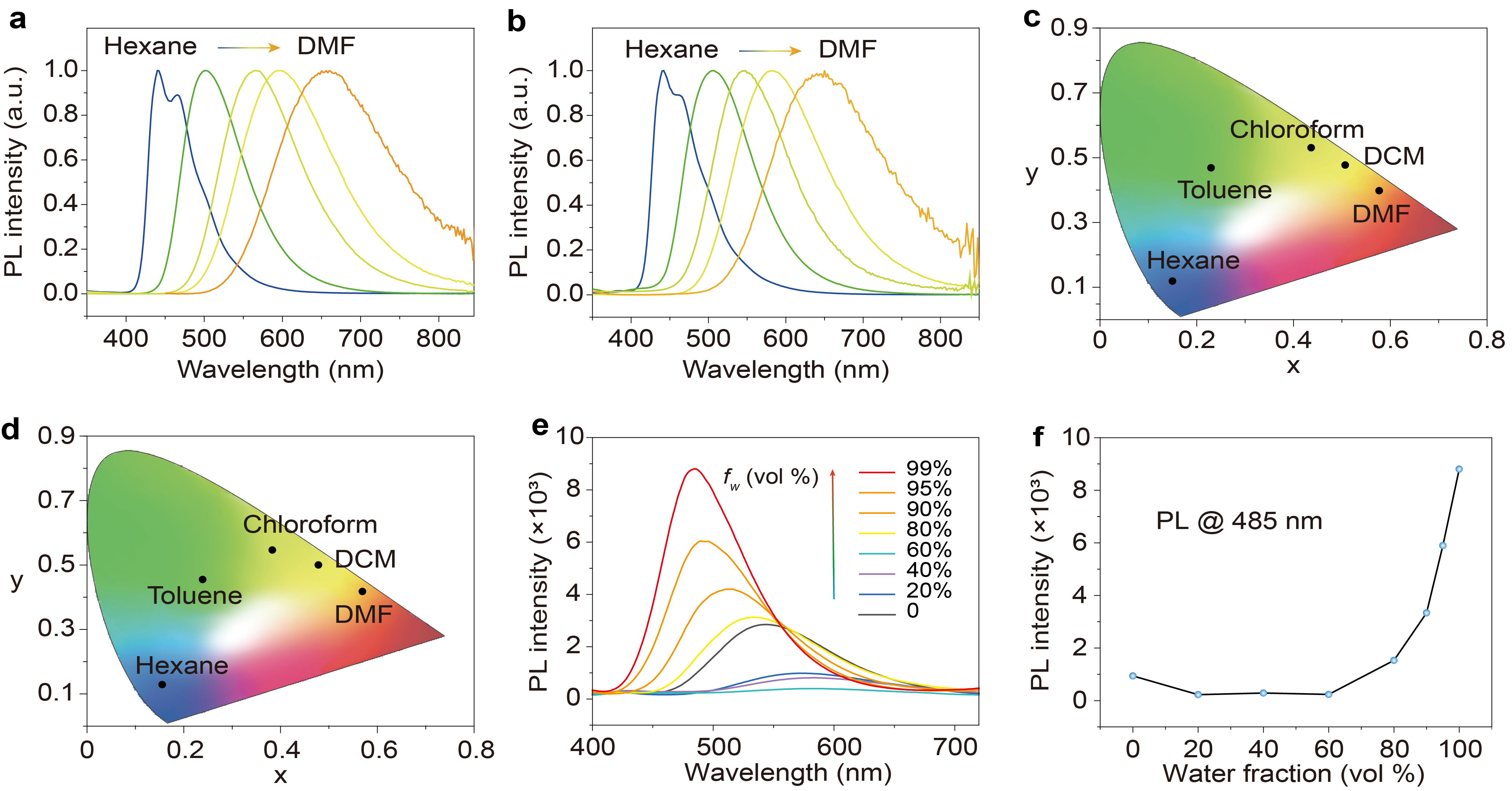


**Figure S8. Photophysical properties in different dilute solutions under excitation of UV light.** Photoluminescence spectra of MAT (**a**) and IMAT (**b**), respectively. Note that the solvents from left to right are in the order of hexane, toluene, chloroform, dichloromethane (DCM), and N,N-dimethylformamide (DMF). The photoluminescence CIE chromaticity coordinate variation of MAT (**c**) and IMAT (**d**) in different solutions. (**e**) Photoluminescence spectra of BMAT in THF/water with varied water fractions. (**f**) Relationship between the BMAT emission intensities at 485 nm with water fractions.


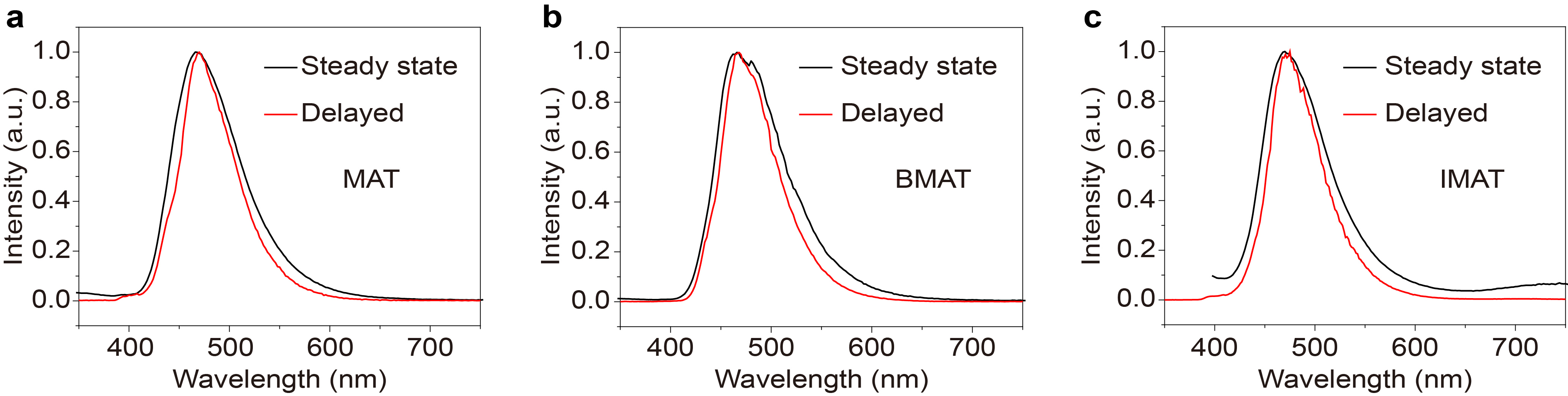


**Figure S9. Steady-state and delayed emission spectra of the scintillators.** Spectra of MAT (**a**), BMAT (**b**), and IMAT (**c**) were tested in toluene solution (*c* = 1×10^-4^ mol/L) at 77 K. The Δ*E*_ST_ values were determined from the maximum emission peaks of steady state (S_1_) and delayed emission (T_1_), respectively.


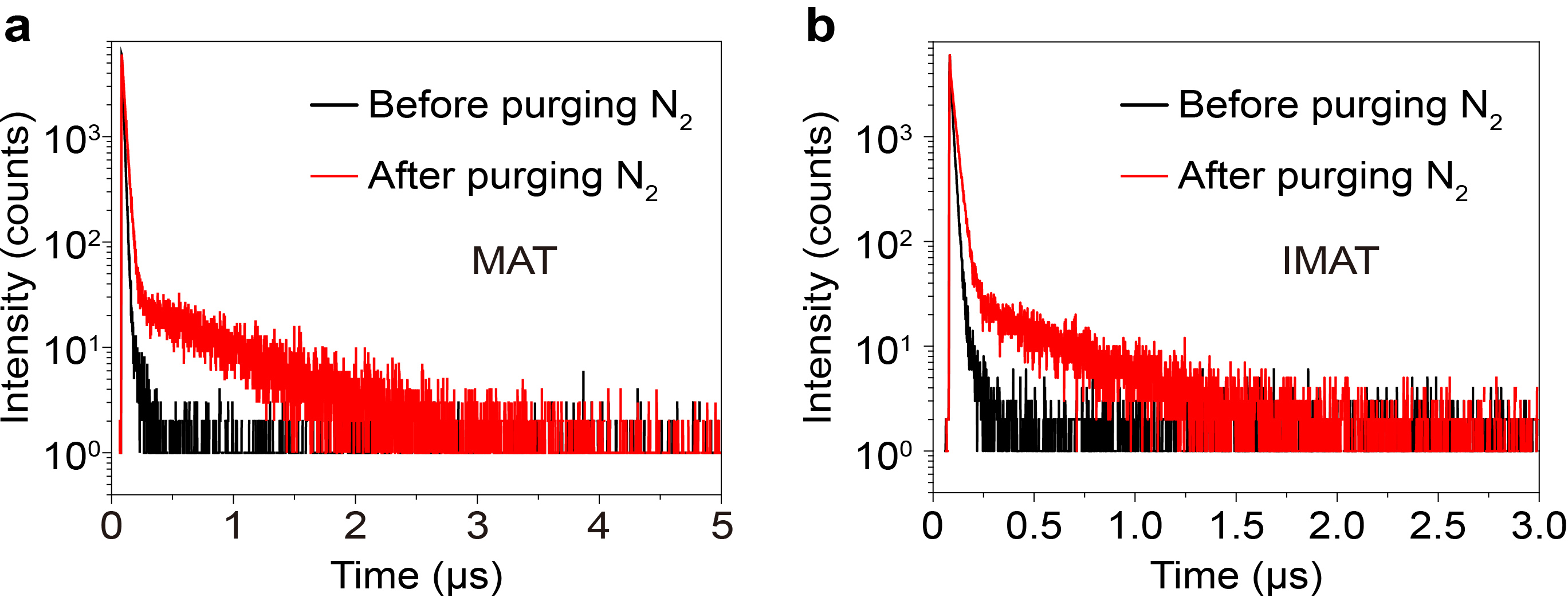


**Figure S10. Room temperature transient photoluminescence decay profiles.** The data of MAT (**a**) and IMAT (**b**) in toluene solution (*c* = 1×10^-4^ mol/L) were recorded before (black) and after (red) purging nitrogen gas for 5 min.

# IV. Density function theory (DFT) calculation.


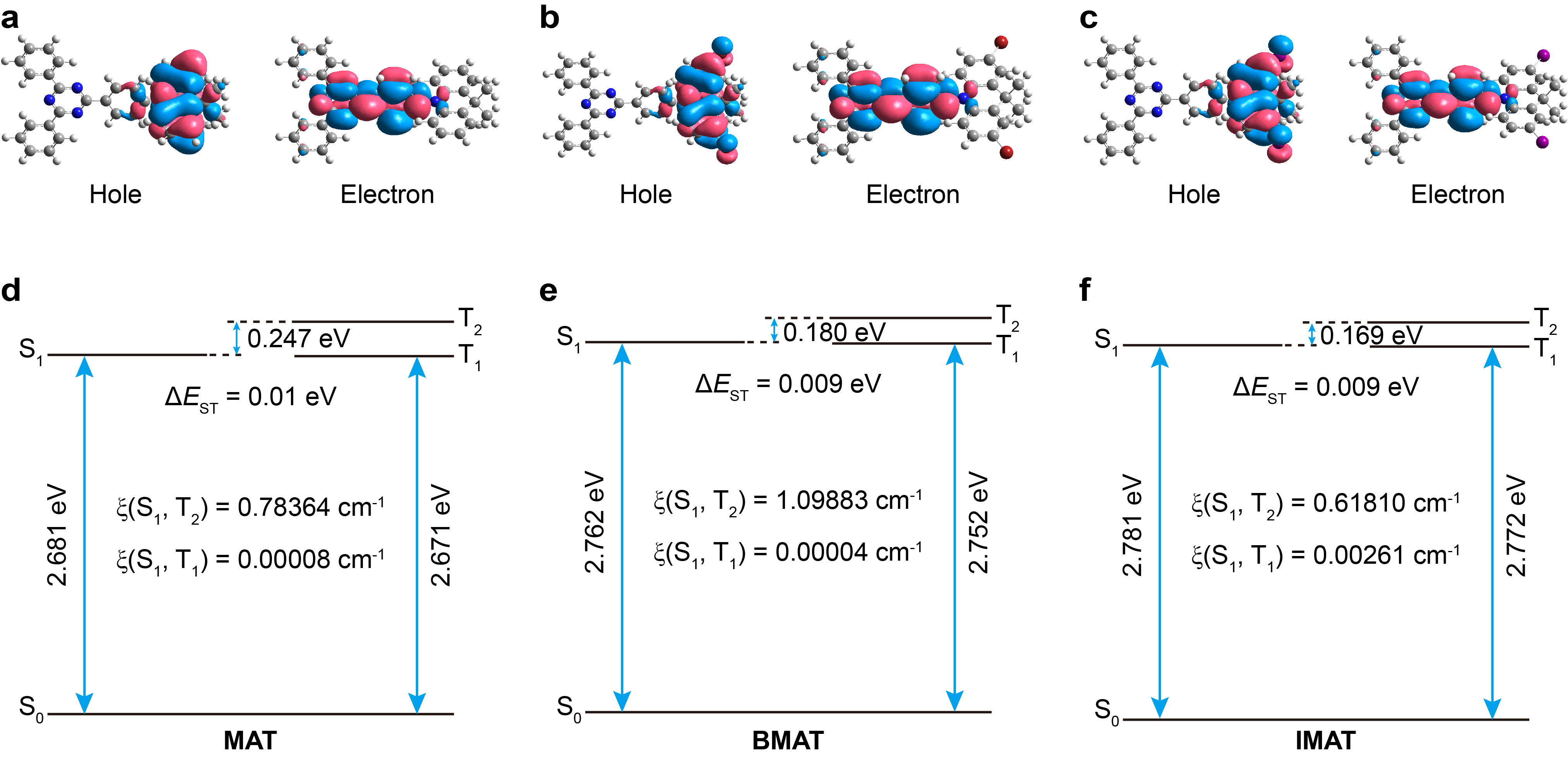


**Figure S11. Electronic configurations and energy level diagrams of the materials.** Natural transition orbitals (NTOs) concerning the lowest singlet excited states (S_1_) of MAT (**a**), BMAT (**b**), and IMAT (**c**). The geometries were optimized at the BMK/def2-SVP level using the Gaussian 09 software package. Energy level diagrams and spin-orbit coupling coefficients (ξ) for MAT (**d**), BMAT (**e**), and IMAT (**f**).


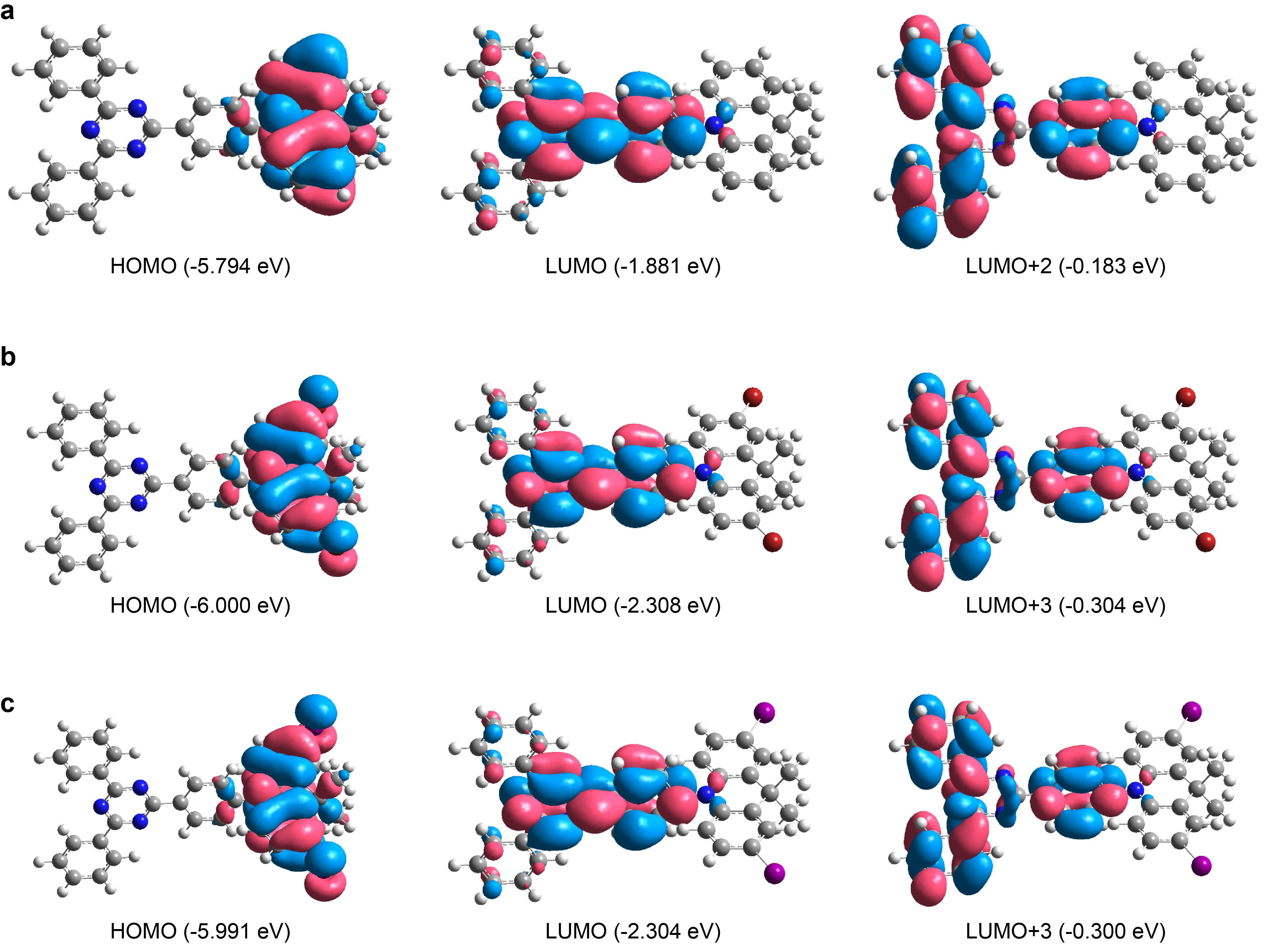


**Figure S12. Theoretical calculation for TADF behaviors of the scintillators.** TD-DFT optimized structures of MAT (**a**), BMAT (**b**), and IMAT (**c**) based on the S_0_ geometry. The theoretically calculated energy levels of related orbitals are added.

**Table S2**. **Molecular orbitals and related proportions of the scintillators based on S_1_.**

| Compound | Molecular orbital | Proportion |
| --- | --- | --- |
| MAT | HOMO🡪LUMO | 94.28% |
|  | HOMO🡪LUMO+2 | 3.44% |
| BMAT | HOMO🡪LUMO | 94.49% |
|  | HOMO🡪LUMO+3 | 3.07% |
| IMAT | HOMO🡪LUMO | 94.26% |
|  | HOMO🡪LUMO+3 | 2.93% |

# V. Detection limit of BMAT scintillator


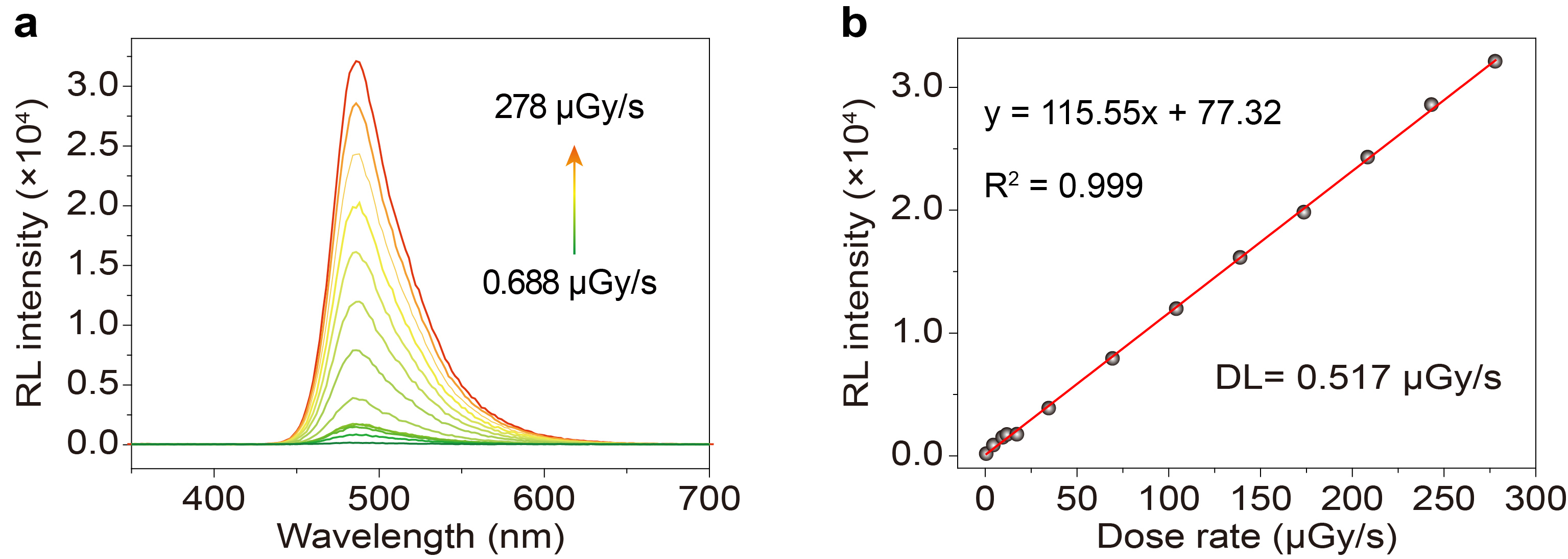


**Figure S13. Evaluating the detection limit of BMAT.** (**a**) Dosage dependent radioluminescence intensity in the range of 0.688 to 278 μGy/s of BMAT. (**b**) Calculation of detection limit (DL) using the 3σ/slope method^S4^.

# VI. Properties of BMAT transparent films


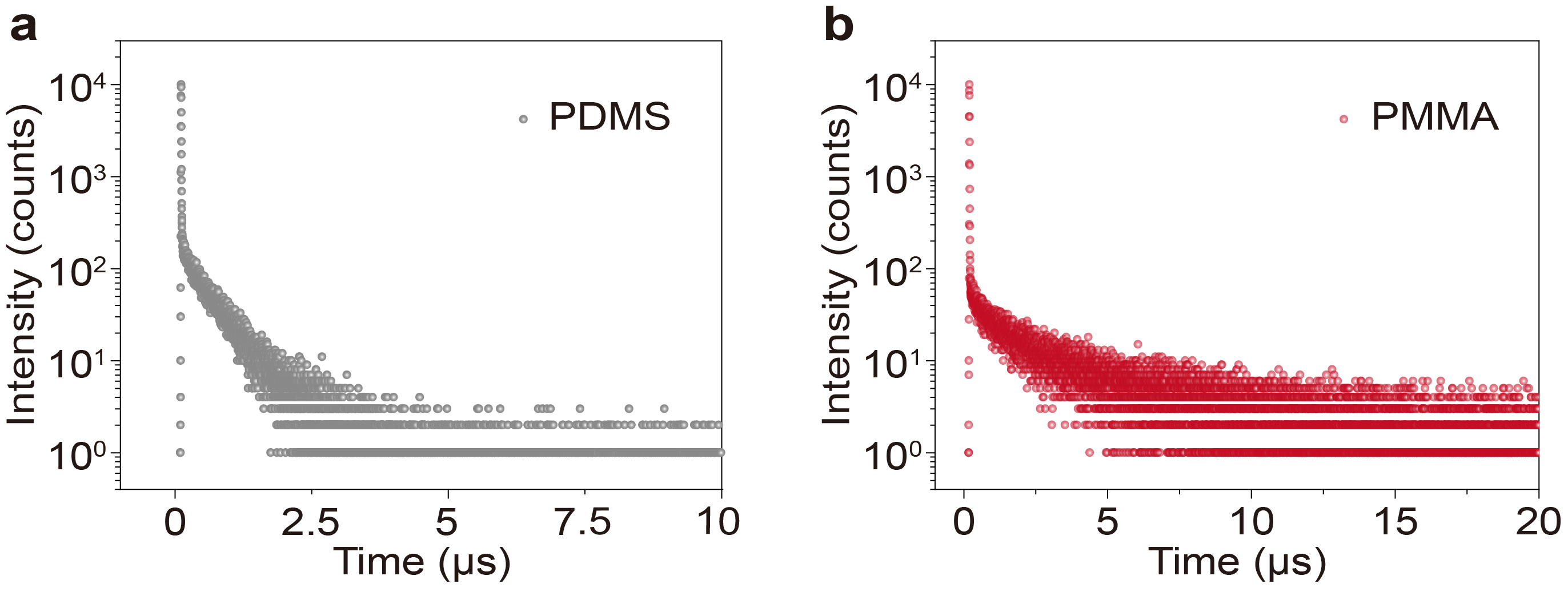


**Figure S14.** **Transient photoluminescence decay curves of BMAT transparent films.** The profiles of BMAT-based PDMS (**a**) and PMMA (**b**) films were recorded, respectively.


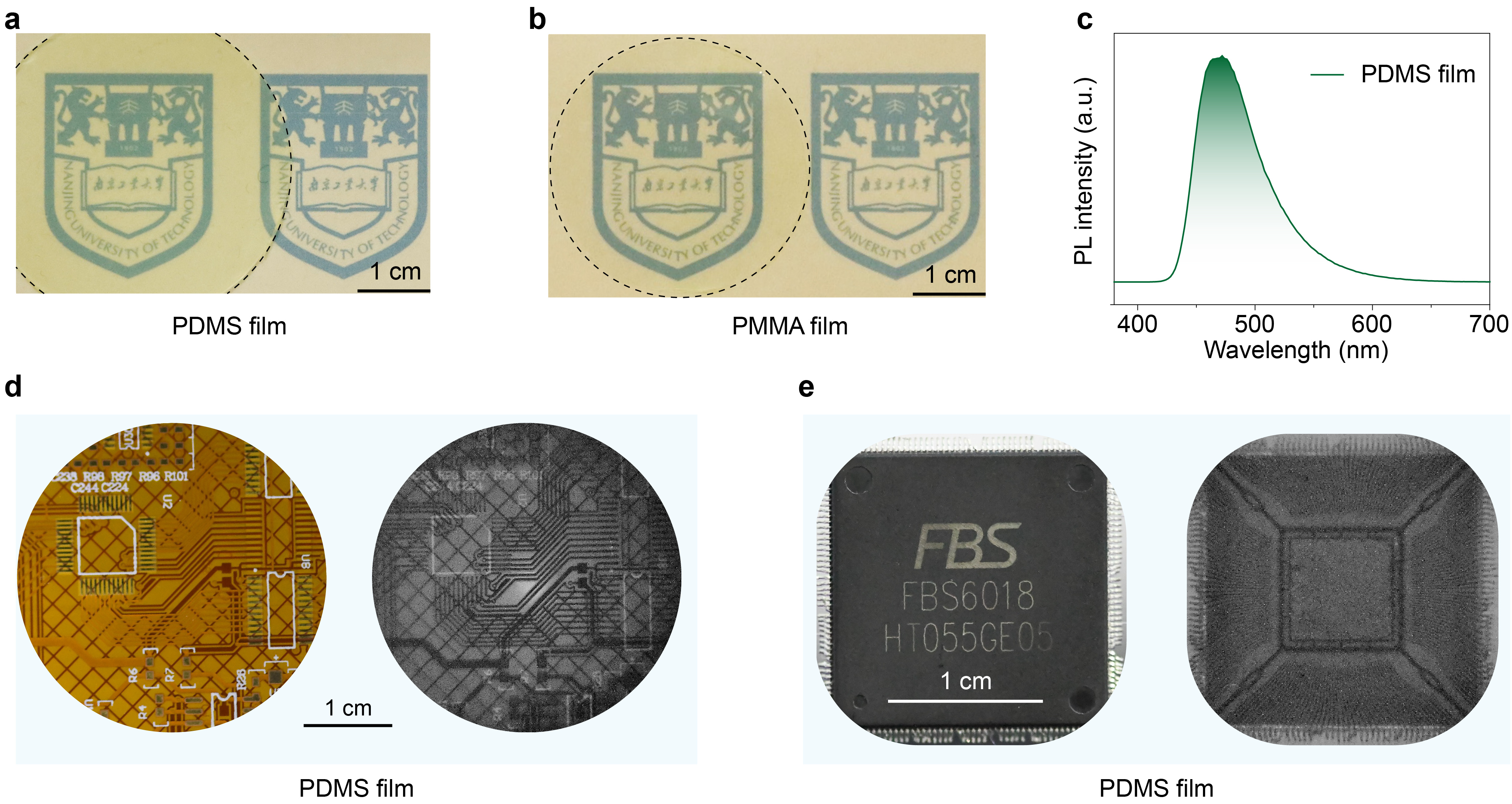


**Figure S15.** **Radiography application of BMAT-based films.** The bright fields of PDMS (**a**) and PMMA (**b**). Dotted circles were added to figure out the boundary of transparent films. (**c**) Photoluminescence spectrum (PL) of the prepared PMMA film. (**d**) Bright- (left) and dark-field (right) photographs of a printed circuit board of PDMS film. (**e**) Bright- (left) and dark-field (right) photographs of a chip of PDMS film.

For PDMS film, we firstly dissolve BMAT in tetrahydrofuran (THF), then we used a silicone elastomer kit (SYLGARD 184) to fabricate the film. Specifically, the two components with a weight ratio of 10:1 were added to the THF solution of BMAT, then the mixture was placed in a container and cured at 323 K for 12 h. While for PMMA film, the mixture of PMMA and BMAT solids were dissolved in dichloromethane (DCM), then the solution was placed in a container and kept at room temperature. Note that the mass ratio between BMAT and matrixes (PMMA and PDMS) was 0.5%.

# VII. References

S1. L. Cui, H. Nomura, Y. Geng, J. Kim, H. Nakanotani, C. Adachi, Controlling Singlet–triplet Energy Splitting for Deep-blue Thermally Activated Delayed Fluorescence Emitters. *Angew. Chem. Int. Ed.* **2017**, 56, 1571.

S2. W. Tsai, M. Huang, W. Lee, Y. Hsu, K. Pan, Y. Huang, H. Ting, M. Sarma, Y. Ho, H. Hu, C. Chen, M. Lee, K. Wong, C. Wu, A Versatile Thermally Activated Delayed Fluorescence Emitter for Both Highly Efficient Doped and Non-doped Organic Light Emitting Devices. *Chem. Commun.* **2015**, 51, 13662.

S3. H. Kim, C. Lee, M. Godumala, S. Choi, S. Park, M. Cho, S. Park, D. Choi, D. Solution-processed Thermally Activated Delayed Fluorescence Organic Light-emitting Diodes Using A New Polymeric Emitter Containing Non-Conjugated Cyclohexane Units. *Polym. Chem.* **2018**, 9, 1318.

S4. G. Long, J. D. Winefordner. Limit of detection. A closer look at the IUPAC definition. *Anal. Chem.* **1983**, 55, 712A.
